# Fractures After Inpatient Falls in a Tertiary Rehabilitation Hospital: Associated Factors, Performance of Fall-Risk Scores, and Orthopaedic Outcomes

**DOI:** 10.3390/jcm15187172

**Published:** 2026-09-15

**Authors:** Fahri Erdi Malkoç, Muhammed Yusuf Afacan, Oğuzhan Yüksel, Dağhan Koyuncu, Furkan Özönder, Nazlı Derya Buğdaycı, Alican Barış

**Affiliations:** 1 Department of Orthopaedics and Traumatology, Istanbul Physical Therapy and Rehabilitation Training and Research Hospital, Istanbul 34180, Türkiye; malkocerdi@gmail.com (F.E.M.); oguzhanyuksel48@gmail.com (O.Y.); daghankoyuncu@gmail.com (D.K.); ozonderfurkan@gmail.com (F.Ö.); dralicanbaris@gmail.com (A.B.); 2 Department of Anatomy, Cerrahpasa Faculty of Medicine, Institute of Graduate Studies, Istanbul University-Cerrahpasa, Istanbul 34098, Türkiye; 3 Department of Physical Therapy and Rehabilitation, Istanbul Physical Therapy and Rehabilitation Training and Research Hospital, Istanbul 34186, Türkiye; deryabugdayci@yahoo.com

**Keywords:** inpatient falls, fall-related fractures, rehabilitation, Morse Fall Scale, Hendrich II Fall Risk Model, bone mineral density, patient safety

## Abstract

**Background/Objectives:** Inpatient falls are an important patient-safety concern in rehabilitation hospitals; however, factors determining whether a fall results in fracture remain poorly understood. This study investigated clinical factors associated with radiographically confirmed fracture after inpatient falls, evaluated the fracture-discrimination performance of the Morse Fall Scale and Hendrich II Fall Risk Model, and characterized the resulting orthopaedic burden. **Methods:** This single-center retrospective cohort study included adults who experienced a documented fall while hospitalized in a tertiary physical medicine and rehabilitation hospital. Patients with and without radiographically confirmed fractures were compared regarding demographic characteristics, comorbidity burden, bone health, continence, mobility, medication exposure, laboratory findings, and fall circumstances. Factors associated with fracture were evaluated using univariable analyses and a parsimonious Firth penalized logistic regression model. Discrimination of the Morse and Hendrich II scores was assessed using receiver operating characteristic analysis. **Results:** Among 91 adult inpatient fall events, 51 (56.0%) resulted in fracture. In unadjusted analyses, patients with fractures had lower lumbar spine T-scores and higher frequencies of urinary and fecal incontinence, whereas age, sex, femoral T-score, mobility status, fall location, and fall mechanism did not differ significantly between groups. In the adjusted Firth model comprising 87 complete cases and 49 fracture events, no variable reached independent statistical significance; lower lumbar T-score (adjusted odds ratio [aOR], 0.74; 95% confidence interval [CI], 0.52–1.05; *p* = 0.073) and urinary incontinence (aOR, 2.30; 95% CI, 0.87–6.12; *p* = 0.082) showed the strongest associations. The Morse and Hendrich II scores demonstrated poor fracture discrimination, with AUCs of 0.372 (95% CI, 0.255–0.489) and 0.411 (95% CI, 0.295–0.526), respectively, without a significant difference between them (*p* = 0.500). Among patients with fractures, 41.2% sustained major fractures, 27.5% underwent surgery, and 11.8% developed complications. **Conclusions:** Among documented inpatient falls in this rehabilitation cohort, fractures were common and imposed a clinically important orthopaedic burden. Conventional fall-risk scores did not distinguish falls resulting in fracture, suggesting that prediction of fall occurrence and prediction of orthopaedic injury represent distinct clinical objectives. Fracture-prevention strategies in rehabilitation settings should therefore complement general fall-risk assessment with targeted evaluation of skeletal and functional vulnerability.

## 1. Introduction

Inpatient falls are among the most common adverse events in hospitals and may lead to physical injury, functional decline, prolonged hospitalization, increased healthcare use, and loss of independence. Fractures are particularly important because they may require immobilization or surgery and may substantially interrupt the rehabilitation process [1,2,3]. In patients with chronic neurological disability, particularly spinal cord injury, fracture management is further complicated by severe sublesional osteoporosis, altered biomechanics, and susceptibility to immobilization-related complications, requiring an individualized balance between operative stabilization and conservative care [4]. Rehabilitation inpatients represent a distinct high-risk population because mobility training, assisted ambulation, transfers, exercise, and toileting expose patients with neurological deficits, impaired balance, cognitive dysfunction, and reduced functional independence to repeated fall opportunities [2,3,5]. Psychoactive and other fall-risk-increasing medications may further increase vulnerability by impairing alertness, coordination, and postural control [1,6].

The Morse Fall Scale and Hendrich II Fall Risk Model are commonly used to estimate the likelihood of falling; however, the performance of inpatient fall-prediction models varies considerably across populations and clinical settings [1,5,7]. Recent guidelines increasingly favor comprehensive, individualized assessment over reliance on scored screening instruments alone [8]. Although patient education, multifactorial interventions, standardized reporting, and coordinated prevention practices are recommended, their implementation remains variable across hospital units [2,9,10,11]. More importantly, conventional fall-risk tools were not developed to determine whether a fall will result in fracture, and the clinical, skeletal, functional, and event-related determinants of fracture among rehabilitation inpatients remain insufficiently characterized.

Accordingly, this study aimed to identify patient-related, clinical, functional, pharmacologic, bone-health, and fall-event characteristics associated with radiographically confirmed fracture after inpatient falls in a tertiary rehabilitation hospital. We also evaluated the ability of the Morse Fall Scale and Hendrich II Fall Risk Model to discriminate between falls with and without fracture and characterized fracture patterns, treatment requirements, complications, and in-hospital outcomes. We hypothesized that skeletal and functional vulnerability would be associated with fracture, whereas conventional fall-risk scores would demonstrate limited fracture discrimination.

## 2. Materials and Methods

This single-center retrospective observational study was conducted in the inpatient physical medicine and rehabilitation (PM&R) wards of a tertiary rehabilitation hospital. The study was designed to identify factors associated with radiographically confirmed fracture after an inpatient fall, evaluate the ability of commonly used fall-risk instruments to discriminate between falls with and without fracture, and describe the orthopaedic characteristics, treatment patterns, and short-term clinical outcomes of patients who sustained fall-related fractures during hospitalization.

The study included two analytic components. The primary analysis compared adult inpatients who experienced a documented fall with those who did and did not sustain a subsequent radiographically confirmed fracture. A secondary fracture-subcohort analysis was performed among patients with fall-related fractures to characterize fracture patterns, treatment strategies, surgical procedures, complications, pressure injuries, and in-hospital outcomes.

The study protocol was reviewed and approved by the Scientific Research Ethics Committee of Istanbul Physical Therapy and Rehabilitation Training and Research Hospital, Istanbul, Türkiye (approval/decision no. 2025-93; 28 November 2025), before data extraction. Owing to the retrospective design, the use of routinely collected clinical and radiological data, and the analysis of de-identified records, the requirement for individual informed consent was waived by the ethics committee. The study was conducted in accordance with the principles of the Declaration of Helsinki and applicable institutional regulations.

All documented inpatient fall events occurring in the PM&R wards between January 2020 and June 2026 were screened for eligibility. The unit of analysis was the documented inpatient fall event. Patients were eligible if they were 18 years of age or older, were hospitalized in a PM&R ward, experienced a documented fall during hospitalization, and had sufficient clinical and radiological documentation to determine whether a fracture had occurred. Patients were excluded if they were younger than 18 years, sustained the fall outside the hospital, experienced high-energy trauma, had a pathological fracture related to malignancy or metastasis, or had insufficient documentation to establish fracture status. The eligible source population consisted of patients admitted to the PM&R wards for indications other than post-fracture recovery; patients admitted specifically for rehabilitation after a pre-existing fracture were not included in the study cohort. Accordingly, the fracture outcome required a new radiographically confirmed fracture occurring after a documented inpatient fall during the current PM&R hospitalization, and fractures present before the index fall were not classified as fall-related outcomes.

A total of 94 inpatient fall events were initially identified. After the exclusion of three fall events involving patients younger than 18 years, the primary cohort comprised 91 documented adult inpatient fall events. Of these events, 51 resulted in a radiographically confirmed fracture and 40 did not. The 51 fracture-associated events constituted the fracture subcohort for descriptive orthopaedic analyses. Four patients had missing lumbar spine T-score data; therefore, the complete-case multivariable model included 87 patients. For patients with more than one recorded fall, only the first eligible fall event was included.

Data were extracted retrospectively from the hospital information management system, inpatient fall-reporting forms, nursing and physician progress notes, quality assurance records, radiology archives, medication records, laboratory databases, orthopaedic consultation notes, operative reports, and discharge documentation. All data were anonymized before analysis. When more than one measurement was available, the most recent value documented before the fall event and consistent with the study-defined variable definition was recorded.

Demographic and baseline clinical variables included age, age category of 65 years or older, sex, Charlson Comorbidity Index, osteoporosis, osteopenia, lumbar spine L1–L4 T-score, and femoral T-score. Total hospital length of stay was evaluated separately as a descriptive hospitalization outcome. Fall-related and functional variables included the time and location of the fall, the activity being performed at the time of the event, fall mechanism, pre-fall mobility status, urinary incontinence, and fecal incontinence. Fall location was categorized as the patient room, another ward area, bed or bedside, bathroom, therapy area or treadmill, and wheelchair or transfer area. Activity at the time of the fall was categorized as ambulation, exercise, or transfer/transport, whereas mobility status was categorized as independent, assisted, walker-dependent, wheelchair-dependent, or bedbound. For mobility coding, “assisted” referred to patients documented as requiring human assistance or supervision for mobility; patients documented as walker-dependent or wheelchair-dependent were coded in those separate categories. Fall mechanism was classified according to the primary event documented in the fall report and clinical record. Because secondary contact with furniture or another object was not consistently available as a separate coded field, a same-level fall could include secondary impact after loss of balance; events in which collision or other external trauma was recorded as the primary mechanism were classified as collision/other trauma. Whether exercise was supervised or unsupervised was not consistently documented and was therefore not analyzed as a separate variable.

Medication exposures of interest included benzodiazepines, sedative or other central nervous system-active medications, and antipsychotics. Laboratory variables included serum 25-hydroxyvitamin D, hemoglobin, sodium, and albumin. For each laboratory variable, the most recent measurement available before the fall event was used.

The primary outcome was a radiographically confirmed fracture after an inpatient fall. A fracture was considered confirmed when it was demonstrated on plain radiography, computed tomography, or magnetic resonance imaging obtained after the fall. The outcome was coded dichotomously as fracture present or fracture absent.

Secondary outcomes included fracture location, fracture severity, operative or non-operative treatment, surgical procedure, in-hospital complications, pressure injury, and total hospital length of stay. For descriptive purposes, fractures were categorized as major or minor using a study-defined clinical classification. Major fractures included fractures of the humerus, vertebra, femoral neck, intertrochanteric femur, subtrochanteric femur, distal femur, and tibial plateau. Other peripheral fractures were classified as minor for descriptive analyses.

The Morse Fall Scale and Hendrich II Fall Risk Model were evaluated as continuous variables. For each instrument, the most recent score documented before the fall event was used.

Among patients with fall-related fractures, additional orthopaedic variables were collected to characterize injury burden and management. These included fracture site, major or minor fracture classification, conservative or operative treatment, type of surgical procedure, recorded in-hospital complications, pressure injury, and total hospital length of stay.

Because only 14 patients underwent surgical treatment, the fracture-subcohort analyses were descriptive. No separate multivariable model was constructed for surgical treatment or postoperative outcomes because the number of events was insufficient to support a reliable adjusted analysis.

### Statistical Analysis

Statistical analyses were performed using IBM SPSS Statistics version 26.0 (IBM Corp., Armonk, NY, USA). Distributional assumptions were evaluated using skewness and kurtosis coefficients; variables with both values within ±3 were considered approximately normally distributed. Normally distributed continuous variables are presented as mean ± standard deviation, non-normally distributed variables as median (interquartile range), and categorical variables as number and percentage. Continuous variables were compared between the fracture and non-fracture groups using the independent-samples *t*-test or Mann–Whitney U test, as appropriate. Variance homogeneity was assessed using Levene’s test, with Welch’s correction applied when necessary. Categorical variables were compared using Pearson’s chi-square test, Fisher’s exact test, or the Fisher–Freeman–Halton exact test with Monte Carlo simulation, according to expected cell frequencies. Factors associated with fracture were initially examined using univariable logistic regression. A clinically informed parsimonious multivariable model was constructed using age, Charlson Comorbidity Index, lumbar spine L1–L4 T-score, urinary incontinence, and sedative/CNS-active medication exposure, with the number of covariates limited according to the available number of fracture events. Because of the modest sample size and potential sparse-data bias, Firth penalized logistic regression was used for the adjusted analysis. Results are reported as odds ratios with 95% confidence intervals. The multivariable analysis was performed using complete cases because lumbar spine T-score data were unavailable for four fall events. The final model therefore included 87 observations and 49 fracture events. No imputation was performed. The discriminatory performance of the Morse Fall Scale and Hendrich II Fall Risk Model for radiographically confirmed fracture was assessed using receiver operating characteristic curve analysis. Areas under the curve were reported with 95% confidence intervals and compared using DeLong’s test. Overall predictive accuracy was assessed using the Brier score. Model calibration was explored using the Hosmer–Lemeshow goodness-of-fit test. Because model development and evaluation were performed in the same cohort, these estimates were considered apparent and exploratory. All tests were two-sided, and *p* < 0.05 was considered statistically significant.

## 3. Results

**Figure 1:**
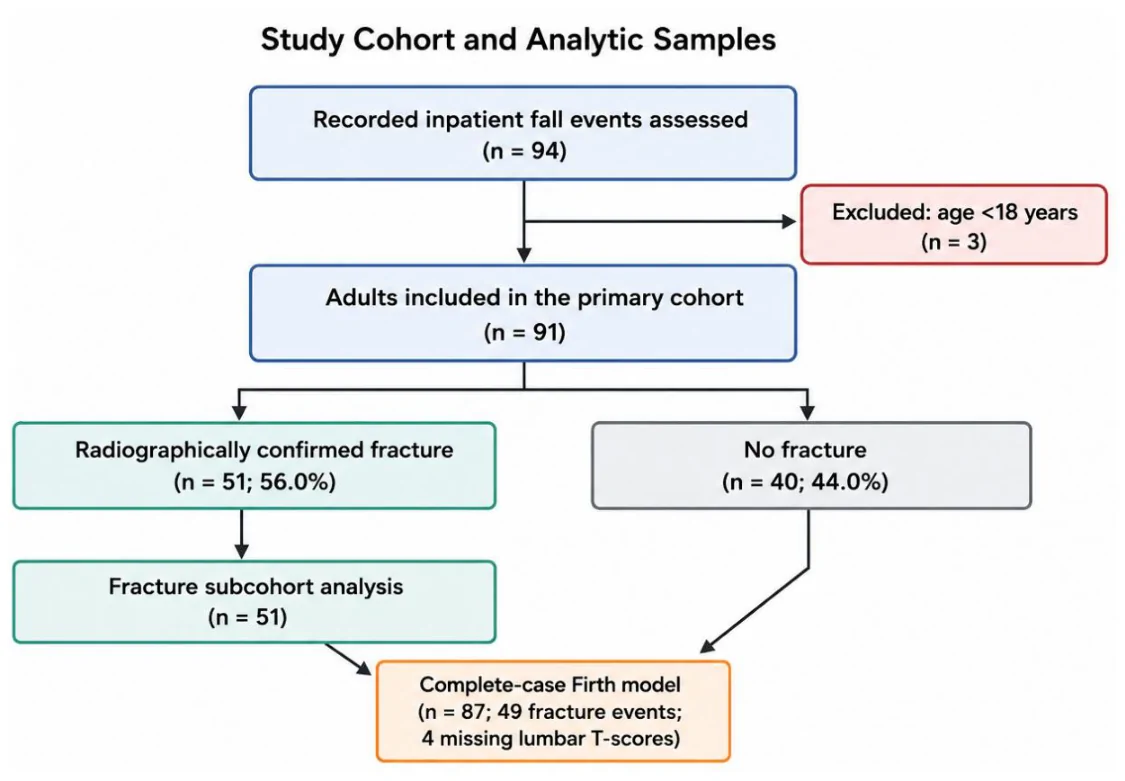
Study cohort and analytic samples. Of 94 recorded inpatient fall events assessed, 3 involving patients younger than 18 years were excluded, leaving 91 adults in the primary cohort. Among these, 51 patients had a radiographically confirmed fracture and comprised the fracture subcohort, whereas 40 had no fracture. The complete-case Firth penalized logistic regression model included 87 patients, of whom 49 had fracture events; 4 patients were excluded from this analysis because lumbar spine L1–L4 T-score data were missing.

Of 94 recorded inpatient fall events, 3 events involving patients younger than 18 years were excluded. The primary cohort therefore comprised 91 documented adult inpatient fall events, of which 51 (56.0%; 95% CI, 45.8–65.8%) resulted in a radiographically confirmed fracture and 40 (44.0%) did not (Figure 1). The mean age was 62.31 ± 14.44 years, and 64 patients (70.3%) were women.

Compared with patients without fracture, those with fractures had a lower Charlson Comorbidity Index (6.00 ± 2.16 vs. 7.28 ± 2.74; *p* = 0.015) and a lower lumbar spine L1–L4 T-score (−1.28 ± 1.22 vs. −0.61 ± 1.42; *p* = 0.021). Urinary incontinence (72.5% vs. 45.0%; *p* = 0.008) and fecal incontinence (31.4% vs. 12.5%; *p* = 0.034) were also more frequent in the fracture group. Sedative or other central nervous system (CNS)-active medication exposure was numerically more common among patients with fracture (54.9% vs. 35.0%; *p* = 0.059), whereas Morse and Hendrich II scores were numerically lower. Age, sex, femoral T-score, osteoporosis, osteopenia, vitamin D, hemoglobin, sodium, and albumin did not differ significantly between groups (Table 1).

Most falls occurred during ambulation (52/91, 57.1%), in either the patient room or another ward area (55/91, 60.4%), and through a same-level mechanism (61/91, 67.0%). The distribution of fall timing, location, activity, mechanism, and pre-fall mobility status was similar between the fracture and non-fracture groups (all *p* ≥ 0.206; Table 2).

In univariable analyses, higher Charlson Comorbidity Index and higher lumbar spine T-score were associated with lower odds of fracture, whereas urinary and fecal incontinence were associated with higher odds. Sedative/CNS-active medication exposure showed a borderline association (Table A1).

**Figure 2:**
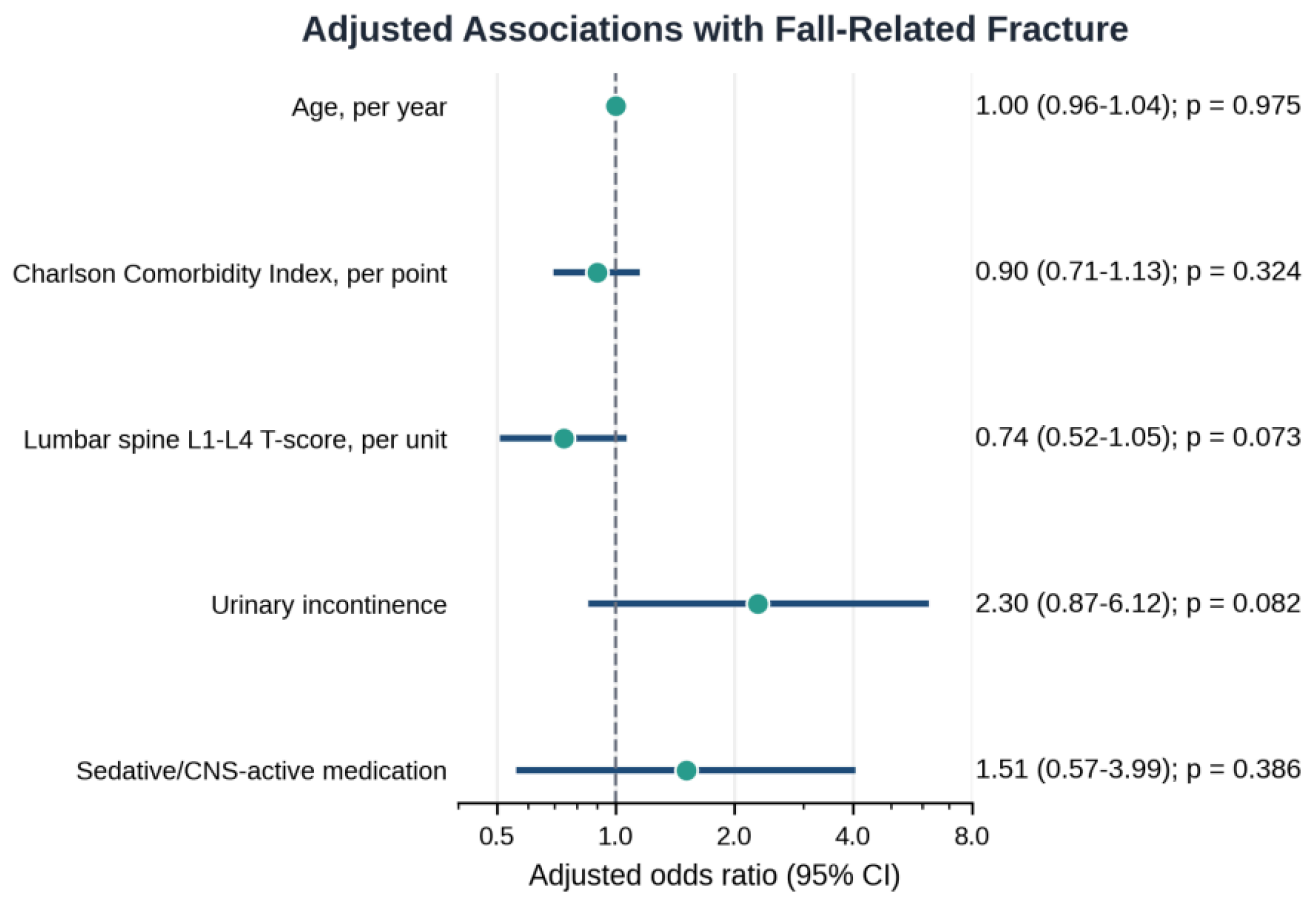
Adjusted associations with radiographically confirmed fracture in the Firth penalized logistic regression model (*n* = 87; 49 fracture events). CNS, central nervous system; CI, confidence interval.

The parsimonious Firth penalized logistic regression model included age, Charlson Comorbidity Index, lumbar spine L1–L4 T-score, urinary incontinence, and sedative/CNS-active medication exposure. The complete-case analysis included 87 patients and 49 fracture events. After adjustment, none of the five covariates met the conventional threshold for statistical significance. The strongest adjusted associations were observed for lumbar spine T-score (adjusted odds ratio [aOR], 0.74 per unit; 95% CI, 0.52–1.05; *p* = 0.073) and urinary incontinence (aOR, 2.30; 95% CI, 0.87–6.12; *p* = 0.082) (Table 3 and Figure 2).

**Figure 3:**
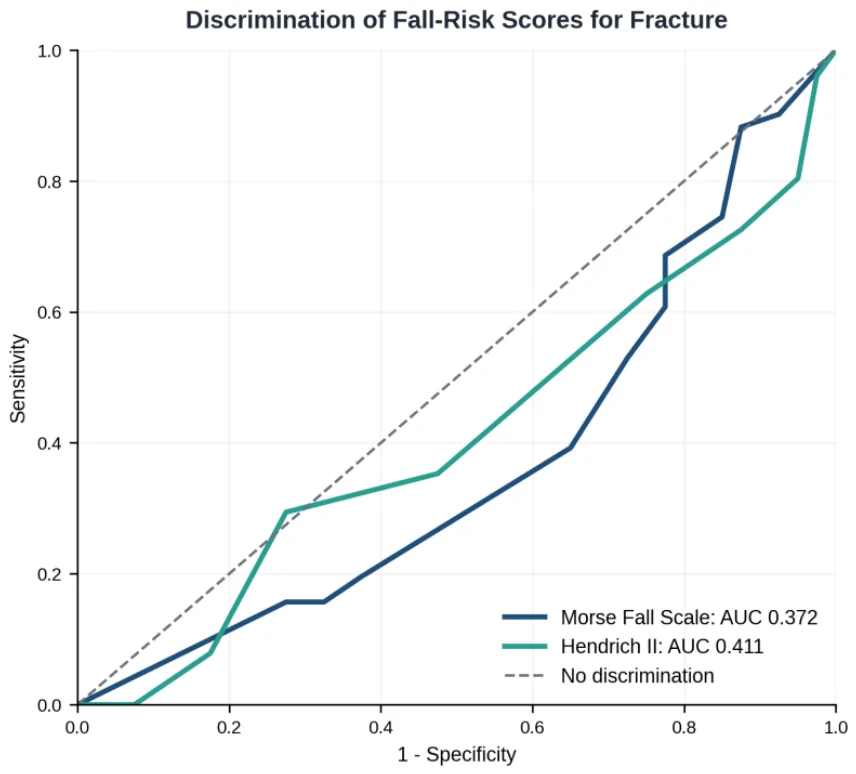
Receiver operating characteristic curves for the Morse Fall Scale and Hendrich II Fall Risk Model as predictors of radiographically confirmed fracture after a documented inpatient fall (*n* = 91). Higher scores were coded as greater predicted risk; AUC values below 0.50 therefore indicate inverse and clinically poor discrimination.

The Morse Fall Scale and Hendrich II Fall Risk Model showed poor discrimination for fracture among patients who had already experienced an inpatient fall. The raw-direction area under the receiver operating characteristic curve (AUC) was 0.372 (95% CI, 0.255–0.489) for the Morse scale and 0.411 (95% CI, 0.295–0.526) for Hendrich II; the difference between the AUCs was not significant (DeLong z = −0.675; *p* = 0.500) (Figure 3). At a Morse threshold of ≥45, sensitivity was 68.6% and specificity was 22.5%; at a Hendrich II threshold of ≥5, sensitivity was 35.3% and specificity was 52.5% (Table 4).

The clinically informed multivariable model had an apparent AUC of 0.717 (95% CI, 0.604–0.830) and a Brier score of 0.208. The Hosmer–Lemeshow test did not indicate gross lack of fit (χ^2^ = 5.22; 3 degrees of freedom; *p* = 0.157). These performance estimates are apparent, in-sample estimates and should therefore be interpreted as exploratory.

Among the 51 patients with fracture, 21 (41.2%; 95% CI, 28.8–54.8%) had a major fracture and 30 (58.8%) had a minor fracture. The most frequent sites were the ankle (10/51, 19.6%), foot or toes (7/51, 13.7%), and humerus (7/51, 13.7%). Thirty-seven patients (72.5%) were managed conservatively, whereas 14 (27.5%; 95% CI, 17.1–40.9%) underwent surgery. Open reduction and internal fixation and hip arthroplasty were the most common operations (4/14 each, 28.6%), followed by proximal femoral nailing (3/14, 21.4%).

**Figure 4:**
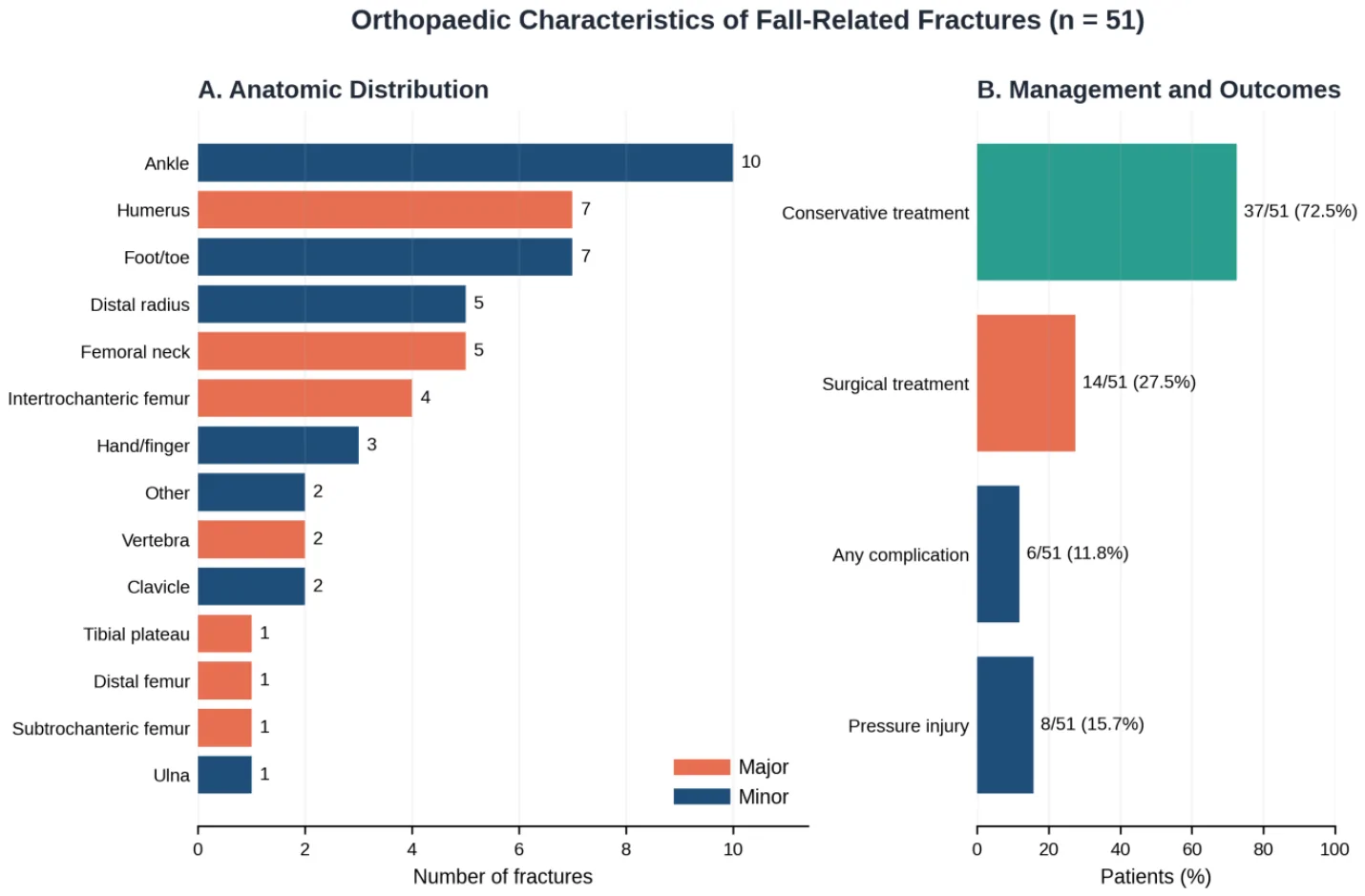
Orthopaedic characteristics of fall-related fractures. (**A**) Anatomic distribution of 51 fractures, categorized as major or minor according to the study-defined clinical classification. (**B**) Treatment and selected adverse outcomes in the fracture subcohort.

Six patients (11.8%; 95% CI, 5.5–23.4%) had a recorded complication; 4 of the 14 surgically treated patients (28.6%) had a complication. Pressure injury was documented in 8 patients (15.7%; 95% CI, 8.2–28.0%). The median total hospital length of stay in the fracture subcohort was 41 days (interquartile range, 26–64 days) (Table 5 and Figure 4). Table A1 shows univariable associations with radiographically confirmed fracture (Appendix A).

## 4. Discussion

This study provides a focused evaluation of fracture as a clinically meaningful consequence of inpatient falls in a tertiary rehabilitation setting. More than half of the included adult fall events resulted in a radiographically confirmed fracture, underscoring the substantial orthopaedic burden associated with falls in this highly vulnerable population. In unadjusted analyses, fracture was associated with a lower lumbar spine L1–L4 T-score, urinary and fecal incontinence, and a lower comorbidity burden, whereas age, sex, femoral T-score, mobility status, fall location, fall mechanism, and most medication and laboratory variables were not significantly different between groups. However, none of the variables included in the parsimonious Firth penalized model remained independently associated with fracture, although lower lumbar spine T-score and urinary incontinence showed the strongest adjusted associations. Importantly, the Morse Fall Scale and Hendrich II Fall Risk Model demonstrated poor discrimination for fracture, indicating that instruments developed to estimate the likelihood of falling may not adequately identify which falls will result in clinically important orthopaedic injury. Taken together, these findings suggest that fracture after an inpatient fall is determined by a complex interaction between skeletal vulnerability and functional impairment and may require a prevention framework distinct from conventional fall-risk assessment alone.

The proportion of fractures in the present cohort was higher than the rates of overall injury or clinically significant orthopaedic harm reported in broader hospital fall registries [1,2,3,12,13]. This difference should be interpreted in the context of the study population and denominator: our cohort consisted exclusively of documented fall events in a tertiary rehabilitation hospital rather than all hospital admissions. Rehabilitation inpatients commonly have neurological impairment, limited protective responses, abnormal loading patterns, and reduced skeletal strength, which may increase the probability that a low-energy fall results in fracture. The substantial proportions of major fractures, surgical treatment, pressure injuries, and recorded complications indicate that the consequences extended beyond radiographic findings and had the potential to disrupt the rehabilitation course. This issue is particularly relevant in patients with chronic neurological disability, in whom immobilization after fracture may accelerate functional decline and increase pressure-related complications [4].

The association between lower lumbar spine T-score and fracture is biologically plausible and supports the contribution of skeletal vulnerability to injury severity. However, femoral T-score and categorical diagnoses of osteoporosis or osteopenia did not differ between groups, and lumbar T-score did not reach conventional statistical significance after adjustment. These findings suggest that fracture susceptibility in rehabilitation inpatients cannot be represented by a single bone-health measure and may reflect the interaction of bone quality, neuromuscular impairment, fall biomechanics, and protective responses. Urinary and fecal incontinence were also more frequent among patients with fracture. Incontinence has previously been linked to inpatient falls through toileting urgency, repeated transfers, impaired mobility, and neurological dysfunction [1,3,12,14,15]. In the present study, however, it may be better interpreted as a marker of functional and neurological vulnerability than as an isolated causal factor. Similarly, the inverse unadjusted association between the Charlson Comorbidity Index and fracture should not be viewed as protective; patients with greater comorbidity may have had lower mobility exposure or closer supervision, and residual confounding remains likely.

CNS-active medication exposure was numerically more frequent in the fracture group but was not independently associated with fracture after adjustment. Previous large inpatient studies have linked sedatives, benzodiazepines, antipsychotics, and several antidepressant classes to the occurrence of falls [6,16]. Our findings do not necessarily contradict that evidence because the present analysis addressed a different conditional outcome: whether a fall resulted in fracture among patients who had already fallen. A medication may increase the probability of falling without independently determining the skeletal consequences of the event. Similarly, fall timing, location, activity, mechanism, and mobility status were not associated with fracture, despite their established relevance to fall occurrence and injury in other hospital cohorts [3,12,13]. This distinction reinforces the need to separate predictors of fall occurrence from predictors of injury after a fall.

The poor fracture discrimination of the Morse and Hendrich II scores represents one of the most clinically relevant findings. Studies in rehabilitation and acute-care populations have reported variable performance of conventional fall-risk tools, with accuracy depending on patient characteristics, timing of assessment, and clinical setting [5,7,14,17,18,19,20]. More recent dynamic and machine learning approaches have explored potentially improved prediction of fall occurrence by integrating neurological, physiological, medication, and longitudinal clinical data [21]. Nevertheless, these models, like conventional scores, primarily predict whether a patient will fall. In our cohort, Morse and Hendrich II scores were numerically lower in patients who sustained fractures, resulting in raw-direction AUC values below 0.50. This inverse pattern should not be interpreted as a protective effect of higher scores; rather, it indicates that once the population was restricted to patients who had already fallen, these instruments provided little information regarding which events would result in fracture. Patient perception and behavioral responses may also differ from standardized risk classifications, further limiting reliance on a score alone [22,23].

The principal novelty of this study lies in reframing the endpoint from fall occurrence to radiographically confirmed fracture after a documented inpatient fall. By simultaneously evaluating skeletal, functional, pharmacologic, and event-related characteristics, the performance of two widely used fall-risk scores, and subsequent orthopaedic management, this study connects the patient-safety literature with clinically meaningful orthopaedic outcomes. Current evidence supports patient education, structured rounding, individualized assessment, and multifactorial prevention rather than reliance on scored screening instruments alone [2,8,9,10,11,24]. Our findings extend this framework by suggesting that rehabilitation services should incorporate fracture-specific considerations, including skeletal fragility, continence, neurological impairment, transfer requirements, and the potential consequences of immobilization. The higher apparent AUC of the multidimensional clinical model supports this concept; however, the model was not internally or externally validated and should not be considered a validated prediction tool. Instead, the findings provide a rationale for larger multicenter studies designed specifically to predict injurious and fracture-producing falls.

This study has several limitations that should be considered when interpreting the findings. First, its retrospective, single-center design limits causal inference and may reduce generalizability to acute-care hospitals, long-term care facilities, and rehabilitation centers with different patient profiles, staffing structures, or fall-prevention practices. Second, the sample size was modest, and the number of fracture events restricted the complexity of multivariable modeling. Although Firth penalized logistic regression was used to reduce small-sample and sparse-data bias, the adjusted estimates remained imprecise, and clinically relevant associations may have been missed because of limited statistical power. Third, the study included only documented inpatient falls; therefore, unreported or incompletely recorded events may have introduced ascertainment and information bias. The magnitude of under-reporting is highly setting-dependent; a 2025 multisite study reported that 29.5% to 90.6% of fall events were absent from institutional self-reporting systems across participating hospitals [25]. An institution-specific under-reporting proportion could not be estimated in the present study because no independent prospective fall-surveillance source was available for cross-validation. In addition, the retrospective records did not consistently distinguish supervised from unsupervised exercise-related falls, and secondary object impact was not available as a separate reliably coded mechanism when the primary event was documented as a same-level fall. Several potentially important variables, including cognitive status, prior falls, frailty, detailed medication burden, orthostatic hypotension, visual impairment, functional scores, environmental hazards, staffing levels, and circumstances immediately preceding the fall, were unavailable or inconsistently documented and could not be incorporated into the final model. Fourth, the Morse and Hendrich II scores were originally developed to predict fall occurrence rather than fracture after a fall; consequently, their poor discrimination in this study should not be interpreted as evidence that they are ineffective for their intended purpose. Fifth, bone health assessment was incomplete, with missing lumbar T-scores in a small proportion of patients, and bone mineral density values may not fully capture skeletal fragility, bone quality, or recent anti-osteoporotic treatment. Sixth, fracture severity and orthopaedic outcomes were evaluated descriptively because the number of surgically treated cases was insufficient for robust adjusted analyses. Finally, the clinical prediction model was developed and evaluated in the same cohort without internal resampling or external validation; therefore, its discrimination and calibration represent apparent performance and should be considered exploratory. In addition, no adjustment for multiple comparisons was applied to the exploratory univariable analyses; therefore, nominal *p* values should be interpreted cautiously. Larger, multicenter prospective studies with standardized fall-event documentation, comprehensive frailty and functional assessment, and independent validation are required to confirm these findings and develop fracture-specific risk stratification tools for rehabilitation inpatients.

## 5. Conclusions

Among documented inpatient falls in this tertiary rehabilitation cohort, fractures were common and imposed a substantial orthopaedic burden. Lower lumbar spine T-score and urinary incontinence showed the strongest associations with fracture, although neither remained independently significant after penalized multivariable adjustment. The Morse Fall Scale and Hendrich II Fall Risk Model demonstrated poor discrimination for fracture, indicating that instruments designed to predict fall occurrence may not identify which falls are most likely to result in clinically important orthopaedic injury. These findings support supplementing conventional fall-risk assessment with fracture-focused evaluation of skeletal fragility, continence, and functional vulnerability. Larger multicenter studies are required to validate these associations and develop fracture-specific risk-stratification approaches for rehabilitation inpatients.

## Figures and Tables

**Table 1:** Demographic, clinical, bone-health, laboratory, medication, and fall-risk characteristics according to fracture status.

Characteristic	Overall (*n* = 91)	Fracture (*n* = 51)	No Fracture (*n* = 40)	*p* Value
Age, years	62.31 ± 14.44	60.14 ± 15.14	65.08 ± 13.17	0.106
Age ≥ 65 years	44 (48.4)	24 (47.1)	20 (50.0)	0.780
Female sex	64 (70.3)	34 (66.7)	30 (75.0)	0.388
Total hospital length of stay, days	54.42 ± 32.51	49.53 ± 30.38	60.65 ± 34.42	0.106
Charlson Comorbidity Index	6.56 ± 2.50	6.00 ± 2.16	7.28 ± 2.74	0.015
Lumbar spine L1–L4 T-score	−0.99 ± 1.34	−1.28 ± 1.22	−0.61 ± 1.42	0.021
Femoral T-score	−1.10 ± 1.84	−1.08 ± 1.94	−1.12 ± 1.72	0.915
Osteoporosis	34 (37.4)	20 (39.2)	14 (35.0)	0.680
Osteopenia	30 (33.0)	20 (39.2)	10 (25.0)	0.152
25-hydroxyvitamin D, ng/mL	13.30 (10.55–21.95)	13.30 (9.25–27.15)	13.15 (11.15–19.30)	0.565
Hemoglobin, g/dL	12.59 ± 1.76	12.30 ± 1.80	12.96 ± 1.66	0.079
Sodium, mmol/L	139.33 ± 3.61	139.20 ± 3.57	139.49 ± 3.71	0.709
Albumin, g/L	39.50 ± 4.04	39.41 ± 4.03	39.61 ± 4.09	0.813
Benzodiazepine exposure	3 (3.3)	3 (5.9)	0 (0.0)	0.253
Sedative/CNS-active medication exposure	42 (46.2)	28 (54.9)	14 (35.0)	0.059
Antipsychotic exposure	5 (5.5)	2 (3.9)	3 (7.5)	0.651
Morse Fall Scale score	60.82 ± 24.95	56.37 ± 24.00	66.50 ± 25.27	0.054
Hendrich II Fall Risk Model score	4.21 ± 2.12	3.84 ± 2.03	4.67 ± 2.15	0.062
Urinary incontinence	55 (60.4)	37 (72.5)	18 (45.0)	0.008
Fecal incontinence	21 (23.1)	16 (31.4)	5 (12.5)	0.034

Values are mean ± standard deviation, median (interquartile range), or *n* (%). Lumbar spine T-score data were available for 87 patients (49 with fracture and 38 without fracture). Vitamin D was compared using the Mann–Whitney U test; other continuous variables were compared using the independent-samples *t*-test. Categorical variables were compared using Pearson chi-square or Fisher exact tests, as appropriate. CNS, central nervous system.

**Table 2:** Fall circumstances and mobility status according to fracture status.

Characteristic	Overall (*n* = 91)	Fracture (*n* = 51)	No Fracture (*n* = 40)	*p* Value
Fall timing				0.421
Morning	48 (52.7)	25 (49.0)	23 (57.5)	
Evening	43 (47.3)	26 (51.0)	17 (42.5)	
Fall location				0.674
Patient room	29 (31.9)	16 (31.4)	13 (32.5)	
Ward area outside patient room	26 (28.6)	17 (33.3)	9 (22.5)	
Bed/bedside	15 (16.5)	6 (11.8)	9 (22.5)	
Bathroom	8 (8.8)	5 (9.8)	3 (7.5)	
Therapy area/treadmill	8 (8.8)	5 (9.8)	3 (7.5)	
Wheelchair/transfer area	5 (5.5)	2 (3.9)	3 (7.5)	
Activity at the time of fall				0.346
Ambulation	52 (57.1)	32 (62.7)	20 (50.0)	
Exercise	21 (23.1)	9 (17.6)	12 (30.0)	
Transfer/transport	18 (19.8)	10 (19.6)	8 (20.0)	
Fall mechanism				0.206
Same-level fall	61 (67.0)	37 (72.5)	24 (60.0)	
Collision/other trauma	30 (33.0)	14 (27.5)	16 (40.0)	
Mobility status				0.740
Independent	8 (8.8)	4 (7.8)	4 (10.0)	
Assisted	50 (54.9)	29 (56.9)	21 (52.5)	
Walker	6 (6.6)	3 (5.9)	3 (7.5)	
Wheelchair	23 (25.3)	14 (27.5)	9 (22.5)	
Bedbound	4 (4.4)	1 (2.0)	3 (7.5)	

Values are *n* (%). Overall *p* values are shown for multi-category variables. Pearson chi-square tests were used for fall timing, activity, and mechanism; Monte Carlo Fisher–Freeman–Halton exact tests were used for fall location and mobility status.

**Table 3:** Firth penalized logistic regression analysis of factors associated with radiographically confirmed fracture.

Variable	Unadjusted OR (95% CI)	*p* Value	Adjusted OR (95% CI)	*p* Value
Age, per year	0.97 (0.94–1.01)	0.110	1.00 (0.96–1.04)	0.975
Charlson Comorbidity Index, per point	0.80 (0.67–0.96)	0.020	0.90 (0.71–1.13)	0.324
Lumbar spine L1–L4 T-score, per unit	0.68 (0.48–0.95)	0.025	0.74 (0.52–1.05)	0.073
Urinary incontinence	3.23 (1.35–7.75)	0.009	2.30 (0.87–6.12)	0.082
Sedative/CNS-active medication exposure	2.26 (0.96–5.30)	0.061	1.51 (0.57–3.99)	0.386

The multivariable complete-case model included 87 patients and 49 fracture events. Odds ratios and confidence intervals were estimated using Firth penalized logistic regression; *p* values are based on penalized likelihood-ratio tests. OR, odds ratio; CI, confidence interval; CNS, central nervous system.

**Table 4:** Discrimination and threshold performance of fall-risk scores and the clinically informed multivariable model.

Instrument/Model	AUC (95% CI)	Threshold	Sensitivity, %	Specificity, %	PPV, %	NPV, %	Brier Score
Morse Fall Scale	0.372 (0.255–0.489)	≥45	68.6	22.5	53.0	36.0	0.236
Morse Fall Scale		≥51	52.9	27.5	48.2	31.4	
Hendrich II Fall Risk Model	0.411 (0.295–0.526)	≥5	35.3	52.5	48.6	38.9	0.237
Clinically informed multivariable model	0.717 (0.604–0.830)	-	-	-	-	-	0.208

Morse and Hendrich II AUCs were calculated using the raw clinical direction, in which higher scores indicate greater risk. Brier scores for the individual scales were derived from logistic probabilities fitted from the continuous score. The clinical model was evaluated in the 87 complete cases; its apparent Hosmer–Lemeshow *p* value was 0.157. AUC, area under the curve; CI, confidence interval; PPV, positive predictive value; NPV, negative predictive value.

**Table 5:** Fracture distribution, treatment, and clinical outcomes in the fracture subcohort (*n* = 51).

Characteristic	*n* (%) or Median (IQR)
Fracture severity	
Major	21 (41.2)
Minor	30 (58.8)
Anatomic location	
Ankle	10 (19.6)
Foot/toe	7 (13.7)
Humerus	7 (13.7)
Femoral neck	5 (9.8)
Distal radius	5 (9.8)
Intertrochanteric femur	4 (7.8)
Hand/finger	3 (5.9)
Clavicle	2 (3.9)
Vertebra	2 (3.9)
Other	2 (3.9)
Ulna	1 (2.0)
Subtrochanteric femur	1 (2.0)
Distal femur	1 (2.0)
Tibial plateau	1 (2.0)
Treatment	
Conservative	37 (72.5)
Surgical	14 (27.5)
Surgical procedure (*n* = 14)	
Open reduction and internal fixation	4 (28.6)
Hip arthroplasty	4 (28.6)
Proximal femoral nail	3 (21.4)
Intramedullary nail	2 (14.3)
Screw fixation	1 (7.1)
Any recorded complication	6 (11.8)
Among surgically treated patients	4/14 (28.6)
Pressure injury	8 (15.7)
Total hospital length of stay, days	41 (26–64)

Major fractures comprised humerus, vertebral, femoral neck, intertrochanteric, subtrochanteric, distal femoral, and tibial plateau fractures. Recorded complications included nonunion (*n* = 3), additional surgery related to perioperative events or nonunion (*n* = 2), and a pressure-injury-related complication (*n* = 1). IQR, interquartile range. Documented pressure injuries and other complication categories were not mutually exclusive.

## Data Availability

The data supporting the findings of this study are not publicly available because they contain potentially identifiable clinical information and are subject to institutional and ethical restrictions. De-identified data may be made available by the corresponding author upon reasonable request, subject to approval by the institutional authorities and Ethics Committee.

## Abbreviations

The following abbreviations are used in this manuscript:

aOR	Adjusted odds ratio
AUC	Area under the receiver operating characteristic curve
CI	Confidence interval
CNS	Central nervous system
IQR	Interquartile range
NPV	Negative predictive value
OR	Odds ratio
PM&R	Physical medicine and rehabilitation
PPV	Positive predictive value
ROC	Receiver operating characteristic

## Appendix A

**Table A1:** Univariable associations with radiographically confirmed fracture.

Variable	Unadjusted OR (95% CI)	*p* Value
Age, years	0.97 (0.94–1.01)	0.110
Age ≥ 65 years	0.89 (0.39–2.04)	0.780
Male sex	1.50 (0.60–3.77)	0.389
Total hospital length of stay, days	0.99 (0.98–1.00)	0.108
Charlson Comorbidity Index	0.80 (0.67–0.96)	0.020
Lumbar spine L1–L4 T-score	0.68 (0.48–0.95)	0.025
Femoral T-score	1.01 (0.81–1.27)	0.914
Osteoporosis	1.20 (0.51–2.83)	0.680
Osteopenia	1.94 (0.78–4.81)	0.155
Urinary incontinence	3.23 (1.35–7.75)	0.009
Fecal incontinence	3.20 (1.06–9.69)	0.040
Sedative/CNS-active medication	2.26 (0.96–5.30)	0.061
Antipsychotic exposure	0.50 (0.08–3.17)	0.465
25-hydroxyvitamin D, per ng/mL	1.01 (0.97–1.04)	0.686
Hemoglobin, per g/dL	0.80 (0.62–1.03)	0.082
Sodium, per mmol/L	0.98 (0.87–1.10)	0.705
Albumin, per g/L	0.99 (0.89–1.09)	0.811
Morse Fall Scale, per point	0.98 (0.97–1.00)	0.057
Hendrich II, per point	0.82 (0.67–1.01)	0.067
Collision/other trauma vs. same-level fall	0.57 (0.23–1.37)	0.208

Odds ratios were estimated using logistic regression; Firth penalization was used where required by sparse data. Benzodiazepine exposure is not shown because all three exposed patients were in the fracture group, producing complete separation; Fisher exact *p* = 0.253. OR, odds ratio; CI, confidence interval; CNS, central nervous system.
